# Joint statement for assessing and managing high blood pressure in children and adolescents: Chapter 2. How to manage high blood pressure in children and adolescents

**DOI:** 10.3389/fped.2023.1140617

**Published:** 2023-04-12

**Authors:** Elke Wühl, Javier Calpe, Dorota Drożdż, Serap Erdine, Fernando Fernandez-Aranda, Adamos Hadjipanayis, Peter F. Hoyer, Augustina Jankauskiene, Susana Jiménez-Murcia, Mieczysław Litwin, Giuseppe Mancia, Artur Mazur, Denes Pall, Tomas Seeman, Manish D. Sinha, Giacomo Simonetti, Stella Stabouli, Empar Lurbe

**Affiliations:** ^1^ Division of Pediatric Nephrology, Center for Pediatric and Adolescent Medicine, Heidelberg University Hospital, Heidelberg, Germany; ^2^ Analog Devices, Inc., Paterna, Spain; ^3^ Department of Pediatric Nephrology and Hypertension, Pediatric Institute, Jagiellonian University Medical College, Cracow, Poland; ^4^ Istanbul University-Cerrahpaşa, Cerrahpaşa Faculty of Medicine, Istanbul, Turkey; ^5^ University Hospital of Bellvitge-IDIBELL and Department of Clinical Sciences, University of Barcelona, Barcelona, Spain; ^6^ CIBER Fisiopatología de Obesidad y Nutrición (CIBEROBN), Instituto de Salud Carlos III, Madrid, Spain; ^7^ School of Medicine, European University Cyprus, Nicosia, Cyprus; ^8^ Department of Paediatrics, Larnaca General Hospital, Larnaca, Cyprus; ^9^ Klinik für Kinderheilkunde II, Zentrum für Kinder und Jugendmedizin, Universitätsklinikum Essen, Universität Duisburg-Essen, Essen, Germany; ^10^ Pediatric Center, Institute of Clinical Medicine, Vilnius University, Vilnius, Lithuania; ^11^ Department of Nephrology and Arterial Hypertension, The Children's Memorial Health Institute, Warsaw, Poland; ^12^ University of Milano-Bicocca, Milano, Italy; ^13^ Institute of Medical Sciences, Medical College, Rzeszów University, Rzeszow, Poland; ^14^ Department of Medical Clinical Pharmacology and Department of Medicine, University of Debrecen, Debrecen, Hungary; ^15^ Division of Pediatric Nephrology, University Children's Hospital, Charles University, Prague, Czech Republic; ^16^ Department of Pediatrics, University Hospital Ostrava, Ostrava, Czech Republic; ^17^ Department of Pediatric Nephrology, Evelina London Children's Hospital, Guys and St Thomas’ NHS Foundation Trust, London, United Kingdom; ^18^ Institute of Pediatrics of Southern Switzerland, Ente Ospedaliero Cantonale (EOC), Bellinzona, Switzerland; ^19^ 1st Department of Pediatrics, Aristotle University of Thessaloniki, Hippokratio General Hospital of Thessaloniki, Thessaloniki, Greece; ^20^ Department of Pediatric, Consorcio Hospital General, University of Valencia, Valencia, Spain

**Keywords:** adolescents, blood pressure, children, hypertension, monitoring, treatment, hypertension-mediated organ damage

## Abstract

The joint statement is a synergistic action between HyperChildNET and the European Academy of Pediatrics about the diagnosis and management of hypertension in youth, based on the European Society of Hypertension Guidelines published in 2016 with the aim to improve its implementation. Arterial hypertension is not only the most important risk factor for cardiovascular morbidity and mortality, but also the most important modifiable risk factor. Early hypertension-mediated organ damage may already occur in childhood. The duration of existing hypertension plays an important role in risk assessment, and structural and functional organ changes may still be reversible or postponed with timely treatment. Therefore, appropriate therapy should be initiated in children as soon as the diagnosis of arterial hypertension has been confirmed and the risk factors for hypertension-mediated organ damage have been thoroughly evaluated. Lifestyle measures should be recommended in all hypertensive children and adolescents, including a healthy diet, regular exercise, and weight loss, if appropriate. If lifestyle changes in patients with primary hypertension do not result in normalization of blood pressure within six to twelve months or if secondary or symptomatic hypertension or hypertension-mediated organ damage is already present, pharmacologic therapy is required. Regular follow-up to assess blood pressure control and hypertension-mediated organ damage and to evaluate adherence and side effects of pharmacologic treatment is required. Timely multidisciplinary evaluation is recommended after the first suspicion of hypertension. A grading system of the clinical evidence is included.

## Introduction

The joint statement is a synergistic action between HyperChildNET and the European Academy of Pediatrics about the diagnosis and management of hypertension in youth, based on the European Society of Hypertension Guidelines published in 2016 with the aim to improve its implementation. The grading system of the clinical evidence reads as follows:

*A* Recommendations are based on randomized trials (or systematic reviews of trials) with high levels of internal validity and statistical precision, provided that the trial results can be directly applied to the patients because of similar clinical characteristics and outcomes have clinical relevance.
*B* Recommendations are based on randomized trials, systematic reviews, or prespecified subgroup analyses that have lower levels of precision, or need to extrapolate from studies in different populations or using validated intermediate/surrogate outcomes.
*C* Recommendations are based on trials that have lower levels of internal validity and/or precision, trials for which non validated surrogate outcomes were used, or results from observational studies.
*D* Recommendations are based on expert opinion alone.

Arterial hypertension (HTN) is the most important risk factor for cardiovascular (CV) morbidity and mortality. The duration of existing HTN also plays a role in risk assessment.

Therefore, just as in adults, appropriate therapy should be initiated in children after the diagnosis of arterial HTN is confirmed and risk factors for hypertension-mediated organ damage (HMOD) are thoroughly assessed.

In all hypertensive children and adolescents, lifestyle interventions incorporating a healthy diet, regular exercise, and weight reduction, if necessary, should be recommended. If this does not lead to normalization of blood pressure (BP) within 6 to 12 months in patients with primary HTN, or if secondary or symptomatic HTN, or HMOD is already present, pharmacological therapy is required. The choice of medication should take into account the cause of HTN. In most cases, blockade of the renin-angiotensin system is recommended as first-line therapy. Combination therapy may be required to optimize BP control, especially in patients with secondary HTN.

Regular follow-up to evaluate BP control and HMOD, and to assess adherence to therapy and side effects of the pharmacological treatment is required.

Early referral to a childhood HTN center, ideally following initial suspicion of HTN, is encouraged to facilitate timely multidisciplinary input including cardiological and nephrological evaluation. The age of transfer to adult care services should be flexible upon patient readiness and specific needs.

## Initial work-up of the hypertensive child

### Patient history

Assessment of patient history should include pre- and postnatal history and a thorough assessment of patient family history including potential risk factors for hypertension, cardiovascular and cerebrovascular disease (1).

In the following materials we present assessment of patient and family history for hypertension and cardiovascular risk factors (Table 1).

**Table 1:** Assessment of patient and family history for hypertension and cardiovascular risk factors.

History	Factors to be assessed
Prenatal history	– Course of mother's pregnancy and possible complications – Results of fetal ultrasound, – Birth weight, – Gestational age at birth – Type of birth – Apgar score
Postnatal history	– Early postnatal course (prematurity, NICU treatment) – Mode of feeding in infancy – Achievement of developmental milestones – Anthropometric variables (height, weight, BMI) – Medical history (incl. symptoms of HTN and HMOD) – History on use of medications – Use of performance enhancing drugs – Smoking – Obstructive sleep apnea – Lifestyle including diet and physical activity
Family history	Information on both parents and grandparents and siblings with focus on – Cardiovascular disease including arterial HTN – Cerebrovascular disease – Obesity – Type 2 diabetes – Chronic kidney disease – Hereditary endocrine diseases or syndromes associated with hypertension

NICU, neonatal intensive care unit; BMI, body mass index; HTN, hypertension; HMOD, hypertension-mediated organ damage.

### Physical examination

Routine pediatric examination with special attention to anthropometric parameters is necessary (1) (Table 2).

**Table 2:** Physical examination of hypertensive children and adolescents for underlying diseases and additional cardiovascular risk factors.

Assessment of	Including
Anthropometry	– Height, weight, waist circumference – Calculation of body mass index
Cardiovascular status	– Blood pressure on upper right arm (see Chapter 1), interpreted by age, sex, and height (link to office BP calculator: https://hyperchildnet.eu/blood-pressure-calculator/) – Pulse – Blood pressure on arms and legs and pulse on lower limbs at first evaluation
Physical status	– General aspects (e.g., growth, edema, habitus, syndromic appearance) – Skin changes (e.g., acanthosis nigricans, café-au-lait spots, neurofibroma) – Eye (e.g., cataract, proptosis) – Abdomen (e.g., mass, hepatosplenomegaly) – Neurological (e.g., cranial nerves, hemiplegia) – Cardiovascular (e.g., femoral pulses, bruit, tachycardia) – Genitalia (e.g., virilisation)

### Laboratory evaluation and ultrasound

Laboratory tests should focus on kidney function and CV risk factors (Table 3).

**Table 3:** Recommended evaluation in children and adolescents with hypertension.

Population	Assessment of
All children and adolescents with elevated blood pressure (Baseline evaluation)	– Blood count and morphology – Ions, including Ca and *P* – Blood gas analysis – Serum creatinine, serum uric acid, total cholesterol, HDL-cholesterol, LDL-cholesterol, triglycerides, glucose, HbA_1C_, thyroid hormones – Urinalysis with spot albumin-to-creatinine ratio – Renal and abdominal ultrasound
Children and adolescents with – ≥ stage 2 HTN, – age <10 years, – Suspected secondary or monogenic hypertension (Usually done in referral centers)	– Renin and aldosterone concentrations – Steroid hormones metabolism – Serum concentrations of meta- and normetanephrine or excretion of catecholamines and their metabolites – Molecular investigations in suspected monogenic HTN – Renal doppler ultrasound

Other imaging studies are usually performed in children in whom despite first step work-up, etiology of arterial HTN remains unestablished and/or in whom secondary HTN is suspected and done in referential centers (1).

### When and how to assess the presence of hypertension-mediated organ damage

The main marker of HMOD is left ventricular hypertrophy (LVH) assessed by echocardiography (ECHO) (Table 4). In children up to 16 years left ventricular mass index (LVMI) should be indexed to height^2.7^ in meters (5).

**Table 4:** Definitions of left ventricular hypertrophy (LVH) by age and sex.

Age	Boys	Girls
≤9 years (2)	LVMI ≥95th percentile	LVMI ≥95th percentile
>9 to 15 years (2, 3)	LVMI >45 g/m^2.7^	LVMI >40 g/m^2.7^
≥16 years (3)	LVMI >50 g/m^2.7^	LVMI >47 g/m^2.7^
≥16 years (4)	LVMI >115 g/m^2^	LVMI >95 g/m^2^

LVMI, left ventricular mass index; Percentile calculation according to Khoury et al. (2).

In Table 5 we present recommendation when and how to assess the presence of hypertension-mediated organ damage.

**Table 5:** Recommendation when and how to assess the presence of hypertension-mediated organ damage.

	Grade
Left ventricular hypertrophy (LVH) should be assessed by echocardiography (ECHO).	B
Electrocardiography is not recommended as a tool for assessment of LVH.	C
Measurement of carotid intima-media thickness (cIMT) and carotid-femoral pulse velocity (PWV) are not obligatory as a first diagnostic step approach to the hypertensive child.	B
cIMT or PWV should be interpreted in relation to appropriate referential values (6–8).	C
Ophthalmoscopy for hypertensive retinopathy is not obligatory at a first diagnostic step. However, in case of severe hypertension/ hypertensive urgency/emergency it may be a useful tool in further therapeutic decisions.	C
Albumin/creatinine ratio in urine should be checked as well as proteinuria and glomerular filtration rate to diagnose kidney damage.	D

## Treatment

### Lifestyle changes

Lifestyle interventions incorporating a dietary component along with exercise or behavioural therapy can lead to improvements in both weight and cardiometabolic factors, including BP in overweight and obese children (1) (Box 1, Table 6).

**BOX 1:** Lifestyle interventions for blood pressure reduction.

Dietary approach	Grade
• *For adolescents with elevated BP a DASH (Dietary Approaches to Stop Hypertension) diet is recommended* (9). • Combined intake of at least 2 servings/day of dairy products and at least 3 servings/day of fruits and vegetables throughout adolescence led to about a 35% lower risk of elevated BP by late adolescence (10). • *Avoiding sugar-sweetened drinks and saturated fat is recommended* • Quantity and quality of dietary fats also influence BP (11). A diet rich in monounsaturated fat resulted in reductions in systolic and diastolic BP (12). Olive oil polyphenols have been associated with several cardiovascular health benefits, especially a BP decreasing effect (13). • Higher sugar sweetened beverage consumption was associated with higher systolic BP (14).	C C C
*Restrictions in salt intake are recommended* (15). The World Health Organization (WHO) recommends a reduction in sodium intake for better control of BP in children aged 2–15 (16). • The European Food Safety Authority (EFSA) Panel on Nutrition considers safe and adequate sodium intake for children (17): ○ 1100 mg/day for children aged 1–3 years ○ 1300 mg/day for children aged 4–6 years ○ 1700mg/day for children aged 7–10 years ○ 2000 mg/day for children aged 11–17 years ○ For infants aged 7–11 months, an adequate intake (AI) of 200 mg/day is proposed based on upwards extrapolation of the estimated sodium intake in exclusively breast-fed infants aged 0–6 months. These recommendations need to be accompanied by a patient education due to difficulties to translate them into real life.	C
**Physical activity**	
• *Regular, daily physical activity is recommended* • Regular physical activity (60 min session, 3 times/week) resulted in a significant reduction of BP (18).	C
**Sedentary lifestyle**	** **
• *For children and adolescents, the sedentary recreational screen time should be limited to 2 h a day, and they should be advised to engage in positive social interactions and experiences* (1). There is evidence of a direct association between sedentary behavior and a high risk of HTN (19).	C
**Sleep Disorders**	
• *Short sleep duration is a risk factor for hypertension*. • There is strong evidence that self-reported short sleep duration, defined by different cutoffs (≤5, ≤6, or ≤7 h), is a risk factor for HTN (20, 21). This has been reported also for several sleep disorders, including sleep apnea and insomnia (21).	C
**Environmental factors**	
• *Exposure to air pollution, environmental noise and outdoor* *temperature affect autonomic cardiovascular regulation, inflammatory pro-coagulative pathways and stress hormones. These mechanisms lead to vascular dysfunction and increases in blood pressure and cardiovascular (CV) events* (22–25).	C

**Table 6:** Lifestyle recommendations to reduce high blood pressure values: goals.

	Grade
– Implement behavioral changes (physical activity and diet) tailored to individual and family characteristics	C
– Involve parents/family as partners in the behavioral change process	D
– Encourage smoke-free environment throughout all stages of life, including discouraging maternal smoking during pregnancy and second-hand smoking	C
– Provide educational support and materials	D
– Establish realistic goals for medical interventions	D
– Develop a health-promoting reward system	D

There is a consensus in the literature that non-pharmacological treatment of adolescent HTN is important because lifestyle modification leads to a reduction in BP (26). However, the results of several trials confirmed that a single factor is rarely capable of controlling an elevated BP and that combined lifestyle interventions (dietary component, physical activity and behavioral therapy) accomplish the most effective reduction in BP. A personalized approach may increase the overall moderate success rate of lifestyle changes.

### Pharmacological treatment

#### When to start

The decision to start pharmacological antihypertensive treatment in a child should not be based on BP level alone but also on the symptoms and comorbidities of the child, the etiology and duration of HTN and the presence of HMOD (Table 7).

**Table 7:** Indications for initiation of pharmacological treatment.

Indication for initiation of pharmacological treatment	Grade
– Symptomatic hypertension	C
– Secondary hypertension	C
– Hypertension-mediated organ damage	C
– Diabetes mellitus type 1 or 2	C
– Primary hypertension > 6 to 12 months refractory to lifestyle modification	D

It should be made clear that, unlike in adults, these recommendations are not based on randomized controlled trials showing that treatment-induced reduction in BP is associated with decreased cardiovascular morbidity and mortality. There are no mortality studies in pediatrics and they will never be performed because cardiovascular mortality in the pediatric population is negligible. However, several uncontrolled studies have shown that antihypertensive drug therapy in children is associated with a reduction in HMOD, such as left ventricular hypertrophy or microalbuminuria.

### Choice of antihypertensive drugs

In contrast to adults, there are no large comparative studies on different classes of antihypertensive drugs in children. One exception is a small short-term study comparing the angiotensin receptor blocker (ARB) valsartan with the angiotensin converting enzyme -inhibitor (ACEi) enalapril, which demonstrated similar antihypertensive efficacy and adverse effects of these two agents blocking the renin-angiotensin-aldosterone system (RAAS) (27). A Cochrane review found 21 randomized controlled trials, usually of short duration and without placebo control groups. Most studies showed BP lowering effects of antihypertensive drugs without severe adverse effects (Grade B) (28). However, no pediatric randomized controlled trials have investigated the efficacy of any antihypertensive drug on the prevention or regression of HMOD.

In the absence of high-quality randomized controlled trials comparing different antihypertensive agents in children, several antihypertensive agents may be considered as first-line drugs [ACEis, ARBs, calcium-channel-blockers (CCBs), ß-blockers, and diuretics] based on the evidence available on HTN in adulthood. An exception is that of children with chronic kidney disease (renoparenchymal HTN) who have been shown to benefit from the antiproteinuric and renoprotective properties of ACE-inhibitors and ARBs.

In addition, certain classes of drugs may be favorable or contraindicated in specific clinical conditions (1) (Table 8). For example, in children with obesity-related HTN, the negative metabolic adverse effects of diuretics and beta-blockers on glucose and lipid metabolism make the metabolic neutral classes of ACE-inhibitors and ARBs potentially preferable.

**Table 8:** Therapeutic approaches.

Present condition	First-line antihypertensive(s)
Chronic kidney disease	ACE-inhibitor or angiotensin-receptor blocker
Diabetes mellitus	ACE-inhibitor or angiotensin-receptor blocker
Coarctation of aorta	Calcium-channel blocker
Obesity-related hypertension	ACE-inhibitor or angiotensin-receptor blocker/Calcium-channel blocker
Posttransplant hypertension	Calcium-channel blocker
Primary hyperaldosteronism	Potassium-sparing diuretic
Congestive heart failure	ß-blocker/ACE-inhibitor or angiotensin-receptor blocker
Microalbuminuria	ACE-inhibitor or angiotensin-receptor blocker
Migraine	Calcium-channel blocker or ß-blocker
Corticosteroid induced hypertension	Thiazide or thiazide-like diuretics

Strict contraceptive measures are required for all girls/women of childbearing age who are prescribed RAAS inhibitors!.

However, in females of childbearing age, RAAS inhibitors should be prescribed only under strict contraceptive measures due a potential teratogenic effect of these drugs.

### Therapeutic approaches in special conditions

#### Combination therapy

Whenever a single agent, prescribed at the maximum recommended or tolerated dose, does not achieve target BP the use of antihypertensive combination therapy is recommended. Combinations of different drug classes with complementary modes of action should be prescribed, taking into account the underlying diseases and comorbidities of the individual patient (Tables 9, 10, Box 2). (Table 8).

**BOX 2:** Goals to achieve in the general hypertensive pediatric population and in diabetic and renal disease.

** **	**GRADE**
**Goals to achieve in the general hypertensive pediatric population**	
In hypertensive children BP should be normalized, i.e., BP levels below the 95th percentile for age, height and sex in children <16 years or below 140/90 mmHg in adolescents 16 years or older should be the first goal. However, attainment of BP levels below 90th percentile is a justifiable recommendation based on expert opinion and results of prospective population studies showing linear relationships between BP levels above 120/80 mmHg in adolescents and higher risk of increased cIMT and cfPWV in the fourth and fifth decade of life (29).	C
**Goals to achieve in diabetic and renal disease**	
In children with CKD BP should be lowered below the 75th percentile. Furthermore, in children with CKD and with proteinuria the goal should be to go below the 50th percentile (1, 30, 31).	A
In children with type 1 or type 2 diabetes mellitus BP should be lowered below the 90th percentile and in those ≥16 years of age below 120/80 mmHg.	D
In children with diabetes mellitus and CKD, the lowest CKD targets should be aimed for.	D

**Table 9:** Exemplary step scheme for combining antihypertensive drug classes.

		GRADE
**Step 1 (Initial therapy)**	**Monotherapy with** • ACEi or ARB • CCB	C
**Step 2**	**Combination Therapy** • ACEi or ARB + CCB • ACEi or ARB + Diuretic	D
**Step 3**	• ACEi or ARB + CCB + Diuretic • ACEi or ARB + CCB + ß-Blocker	D

ACEi, angiotensin converting enzyme inhibitor; ARB, angiotensin receptor blocker; CCB, calcium channel blocker; Strict contraceptive measures are required for all girls/women of childbearing age who are prescribed RAAS inhibitors!.

**Table 10:** Principles of antihypertensive therapy in children with diabetes mellitus type 1 or 2 (grade C).

BLOOD PRESSURE THRESHOLDS	TREATMENT
BP >90th percentile in children and adolescents <16 years of age and/or >130/80 mmHg in adolescents 16 years of age or older	Non-pharmacological treatment (weight loss; healthy diet; regular exercise)
BP >90th percentile despite 6 months of non-pharmacological treatment	add ACEi/ARB
BP >95th percentile in children and adolescents <16 years of age and/or above 140/90 mmHg in adolescents 16 years of age or older	Non-pharmacological therapy + ACEI/ARB (started immediately, combined antihypertensive treatment possible)

It is recommended to monitor the effects of treatment with home blood pressure monitoring and/or ABPM.

ACEi, Angiotensin converting enzyme inhibitor; ARB, Angiotensin receptor blocker; Strict contraceptive measures are required for all women of childbearing age who are prescribed RAAS inhibitors! (See Chapter 1; link to office BP calculator: https://hyperchildnet.eu/blood-pressure-calculator/ and to ABPM calculator: https://hyperchildnet.eu/ambulatory-calculator/.

## Motivation strategies for patients and caregivers

### Essential information

As in many other chronic diseases (32), patient adherence, the degree to which patients follow the recommendations of their health professionals, is a salient outcome of the process of care and, treatment nonadherence is a major problem in the management of hypertension. Therefore, it is important to introduce motivation strategies for patients and caregivers to increase adherence to treatment (33).

### Motivation to change

Motivation to change includes the constructs that must arise from the person him/herself and that are not imposed by third parties. It is an internal state, which fluctuates according to external factors. It has a series of phases, through which the person may undergo, ranging from the decision not to change (by considering only the positive/gratifying aspects of the harmful behavior), to a complete and stable change of habits in favor of a healthier lifestyle such as an adequate diet, physical exercise, giving up tobacco, alcohol, or even increasing adherence to pharmacological treatment (34).

### Motivational style

A motivational style has to be followed, on the part of the family and/or physician, by asking openly these types of questions: asking to know how the person feels (with unhealthy habits), what worries he/she has, what doubts he/she has regarding the new habits to change, why do they think it might be important to change his/her new habits, asking about his/her opinion about potential positive consequences of doing so, helping him/her to explore his/her ambivalences and discrepancies. The most important tool to achieve this motivational style is empathy, although others may be active and reflective listening (showing genuine and sincere interest in understanding their concerns and doubts in relation to the change), honesty, not judging or criticizing, being willing to go at the individual's pace, promoting self-efficacy (see Boxs 3, 4 and Table 11) (35, 36).

**BOX 3:** Motivational Interview.

Motivational interview
• Important points to keep in mind in a **motivational interview** with patients and carers: • Direct, patient-centered style of assistance to elicit behavioral change, helping to explore and resolve ambivalences. • Motivation for change comes from the patient. It is not imposed. • The goal of the interview is to achieve intrinsic motivation for change (vs. extrinsic motivation), is not to impose the views of the family or the healthcare provider. • Acceptance of ambivalence as a normal part of the human experience. • Uses specific strategies to create motivation for change. • The practitioner focuses on encouraging the patient/carer to express his or her concerns. • The practitioner focuses on encouraging the patient/carer to express the positive impact (short and middle term) that will result from changing that behaviour and following the HCP recommendations • Use open questions.

**BOX 4:** Identifications of resistances by the patient/family member.

Identifications of resistances by the patient/family member
• Arguing (challenging, devaluing, questioning the pediatrician's authority). • Interrupting (cutting or not letting finish). • Denying (minimizing, excusing, blaming others, showing pessimistic attitude). • Ignoring (not attending, not responding, changing the subject, looking the other way…).

**Table 11:** Motivation style and recommendations .

Empathy	Generate discrepancy	To avoid arguing	Promoting self-efficacy	Change the resistance
**Attitude of acceptance:** • Acceptance of the person as he/she is. • Acceptance of her ambivalence • Non-judgment of the person • Use of reflective listening (desire to understand the patient's/carer's points of view)	• Create and amplify a discrepancy between the consequences of the current behavior and the most important objectives to be achieved. • Analyze the consequences of current behavior in the medium-long term, which prevent the achievement of personal goals. • Let the patient and/or carer, if desired, express the reasons for wanting to change.	• Not convincing the patient/ carer that he/she has a problem and needs to change it when he/she may not want to. • Avoid arguing, as they usually generate more resistances. • If resistances appear, it is up to the HCP/pediatrician to change the strategy to be used.	• Increase the patient's/ carer's personal belief in performing a given behavior (“I can do it”, “I can get it”, “What's the harm in trying”?) • Emphasize the need for personal responsibility for change.	• Identification of resistance • Avoid arguing • New points of view are suggested, not imposed.

## Suggested schedule for follow-up examinations

Follow-up of hypertensive individuals is based on the underlying etiology of the high BP condition, presence of co-morbidities, severity of HTN, presence of HMOD and patient's knowledge and understanding of the rationale of the HTN management (Table 12) (1).

**Table 12:** Follow-up evaluation for HMOD in children with hypertension.

Examination	Condition	Interval
Echocardiography	Well-controlled HTN and no HMOD	Every 18 to 24 months
	Suboptimal BP control	Every 6 to 12 months
	Left ventricular hypertrophy	Every 6 to 12 months
Fundoscopy	Well-controlled HTN and no HMOD	Every 18 to 24 months
	Uncontrolled hypertension	At least annually
	Evident Retinopathy	At least annually

In the majority of patients with primary HTN, especially those advised non-pharmacological lifestyle changes, infrequent but regular, follow-up is advisable. In contrast, in those whose management includes both pharmacological treatment and lifestyle changes, a more frequent regular follow-up is needed. Home BP monitoring can facilitate the management of HTN. Telemedicine could additionally be helpful. Ideally, in those with chronic kidney disease or diabetes annual ABPM should be recommended to rule out BP control limited to office BP a condition that in adults is termed MUCH (masked uncontrolled HTN). Accessibility to appropriate resources to facilitate this is a key factor.

Patients with well-controlled HTN and no HMOD should be monitored in regular intervals to rule out *de novo* HMOD, and to assess adherence to therapy and side effects of the pharmacological treatment (1).

Optimal BP control over a prolonged period of time may justify a reduction of the number and dose of antihypertensive drugs. Healthy lifestyle changes should be maintained, the reduction of pharmacological therapy should be made gradually and the patient should frequently be monitored (at 3 to 6 months intervals) because of the risk of re-emergence of HTN (1).

## Long-Term consequences of childhood hypertension

Hypertension remains the main modifiable risk factor for the risk of cardiovascular disease and death, and cardiac and vascular damage by an elevated BP begins in childhood.

Early organ damage occurs in the form of left ventricular hypertrophy, thickening and stiffening of the vascular wall, albuminuria, and cognitive dysfunction. HTN and other cardiometabolic factors cause endothelial dysfunction and are responsible for the accelerated process of atherosclerosis. At this stage, organ structural and functional changes may still be reversible or postponed with appropriate treatment.

In recent years, studies have shown both tracking of high BP into adulthood and increased cardiovascular risk in young adults with childhood onset of HTN. High normal BP and hypertensive trajectory groups have worse cardiovascular outcomes by early midlife. Higher body mass index and cigarette smoking are associated with increased BP values across trajectories, particularly in the higher BP groups that in childhood predict adult cardiovascular risk (37).

In a prospective cohort study, childhood risk factors (higher body-mass index, systolic BP, total cholesterol level, triglyceride level, and youth smoking) and the change in the combined-risk z score between childhood and adulthood were associated with cardiovascular events in midlife (38).

The goal of antihypertensive treatment is to reduce the global risk of cardiovascular events. Recognition of risk factors and early cardiovascular damage, at a time when these processes are still reversible, and development of prevention strategies are key factors for the reduction of cardiovascular morbidity and mortality in the general population (39). Long-term follow-up multicenter studies are necessary to assess cardiovascular risk stratification in children and adolescents with HTN and to develop standards for diagnostic and treatment procedures (1, 37–39).

## Criteria to refer patients to pediatric specialists

Pediatric specialists for arterial HTN, usually pediatric nephrologists or cardiologists, should be involved in the management of a persistently elevated office BP in an asymptomatic healthy child, i.e., elevated BP values over at least three separate visits (Table 13) (1).

**Table 13:** Recommended responsibility for hypertension work-up and management in children and adolescents.

Examination/test/procedure	Pediatrician/family doctor	Hypertension specialist
Screening for high blood pressure	**x**	** **
Baseline investigations	**x**	** **
ABPM	** **	**x**
Evaluation for etiology of HTN	** **	**x**
Evaluation for HMOD	** **	**x**
Non-pharmacological treatment	**x**	**x**
Pharmacological treatment (Decision making on treatment and on drug class(es)	** **	**x**

Patients should be referred to specialist management earlier (after one or two visits with a BP elevation) if stage 2 HTN is present or HTN is symptomatic. Intervals between visits should be judged on a clinical basis (1).

We encourage early referral to a childhood HTN center, ideally following initial suspicion of HTN, to facilitate timely multidisciplinary input including cardiological and nephrological evaluation. This should encourage Children's Hospitals in Europe to organize expert HTN services which will provide an opportunity for specialist care to work together with family physicians, pediatricians, the hypertensive child and their family, with a greater chance for optimal HTN management.

## Reaching the age for transition to adult units. How to proceed

### Essential information

Transition refers to the process of moving from pediatric to adult care services beginning in adolescence and continuing into early adulthood (Box 5). This should be done according to preplanned strategies, with the overarching goals to develop patient's readiness, increase the young people' s level of responsibility for their own health care management, engage and enhance the understanding of the conditions they are living with, and build the skills needed to navigate the adult care system. Family support and both patient and family willingness to transfer are also key elements to be considered during transition (40, 41).

**BOX 5:** Indications for the transition from pediatric to adult care services.

	Grade*
• A **structured health history** with information on the etiology of HTN, disease course, comorbidities, previous and current treatment as well as report on physiological issues and treatment adherence should accompany transfer of hypertensive youth to adult care.	D
• **Close follow-up** for adherence to treatment and possible adverse outcomes before and after transfer to adult health units requires a careful management and coordination between pediatric and adult health care providers.	D
• **The age of transfer** to adult care services should be flexible upon patient readiness and specific needs.	D

In pediatric patients with early onset of HTN the transition process may start at the age of 12–14 years, gradually introducing an individualized transition plan during adolescence (40–42) based also on the presence of HMOD and/or comorbidities. The adoption of adult thresholds for the diagnosis of HTN in adolescents ≥16 years old by the ESH 2016 guidelines (1), enable smooth transition to adult health care.

### Present challenges

• *Little guidance* is provided in the literature, with low evidence on the effectiveness of any specific intervention on transition to improve transfer to adult health care (43).
• *Involving primary care practitioners in transition*. Very little is known about the impact and role of primary health providers in supporting the transition of youth with health care needs. Community services and primary care teams tend to provide holistic care and are effective for integrating social determinants of health.

## Conclusions

Arterial hypertension is the most important modifiable risk factor for cardiovascular morbidity and mortality. Therefore, appropriate antihypertensive therapy including lifestyle measures and pharmacologic treatment should be initiated in children as soon as the diagnosis of arterial hypertension has been confirmed and the risk factors for hypertension-induced organ damage have been thoroughly evaluated. Early referral to a pediatric hypertension center, ideally after the first suspicion of hypertension, is recommended to allow timely multidisciplinary evaluation, including workup by pediatric cardiologists and nephrologists, and initiation of antihypertensive treatment.
